# Functional Yogurt: Enhancing Color Stability, Functional Quality, and Storage Life With Malabar Spinach ( *Basella alba* L.) Fruit

**DOI:** 10.1002/fsn3.70710

**Published:** 2025-07-29

**Authors:** Md. Akhtaruzzaman, Tanjim Ahmed, Md. Rakibul Islam, Sharmin Akther, Md. Sajib Al Reza

**Affiliations:** ^1^ Department of Food Engineering Jashore University of Science and Technology (JUST) Jashore Bangladesh; ^2^ Department of Food Processing and Preservation Hajee Mohammad Danesh Science and Technology University (HSTU) Dinajpur Bangladesh; ^3^ Department of Food Technology and Nutritional Science Mawlana Bhashani Science and Technology University (MBSTU) Tangail Bangladesh

**Keywords:** betalain, functional properties, natural colorant, plant‐based food, ultrasonic extraction

## Abstract

Malabar spinach ( *Basella alba* L.) is a leafy green vegetable rich in betalains, and is found in its fruit. It is also rich in bioactive and antioxidant compounds. This study investigated the potential use of malabar spinach fruit extract (MSFE) at four concentrations (T_0_ = 0 μL (control), T_1_ = 500 μL, T_2_ = 1000 μL, and T_3_ = 2000 μL) in yogurt as a natural colorant during 21 days of refrigerated storage. The pH and syneresis of the MSFE‐containing yogurt significantly decreased (*p* < 0.05). Moreover, the total soluble solids, viscosity, and titratable acidity increased (*p* < 0.05) in a dose‐dependent manner and maintained a similar pattern upon storage. Furthermore, compared with the control yogurt, the MSFE‐treated yogurt exhibited higher total phenolic content (29.97 mg GAE/100 g DM, T_3_) and total flavonoid content (6.72 mg QE/100 g DM, T_3_) as well as increased antioxidant activity (15.47%, T_3_). Higher MSFE levels significantly reduced (*p* < 0.05) the initial total plate count from 520.5 × 10^6^ CFU/g (T_1_) to 118.7 × 10^6^ CFU/g (T_3_). However, bacterial proliferation led to an increase in the total plate count during storage. MSFE significantly enhanced (*p* < 0.05) yogurt color properties, with T_3_ resulting in the highest betalain content (2.776 mg/L). The betalain content slightly decreased (*p* < 0.05) after 14 days but regenerated by the end of the storage period. Nevertheless, MSFE exhibited acceptable color stability over a wide pH range during storage. These findings suggest that MSFE is a viable natural colorant for yogurt, offering improved nutrition and consumer appeal, which was further confirmed by principal component analysis (PCA).

## Graphical abstract

This study investigated the impact of Malabar spinach fruit extract (MSFE) on yogurt quality, revealing that 2000 μL MSFE enhanced physicochemical properties like viscosity, acidity, and soluble solids while reducing syneresis. MSFE also boosted antioxidant activity, phenolic and flavonoid content, and improved color stability while suppressing bacterial growth. Sensory analysis and PCA confirmed MSFE's role in elevating yogurt quality and consumer acceptance.

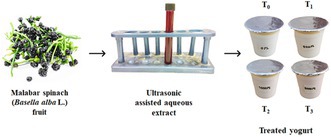

## 1 Introduction

Natural pigments with bioactive functions have become more popular due to the possible health hazards associated with synthetic ingredients (Nabi et al. 2023). Furthermore, recent advancements in food manufacturing and consumption have sparked a demand for novel food additives. These additives may appeal to consumers by serving hedonic purposes such as providing color and flavor, improving food characteristics through preservation with antimicrobials, or offering beneficial attributes such as antioxidants and prebiotics (Schneider‐Teixeira et al. 2022). Color is the first feature of the product observed by the consumer and has a critical influence on either the acceptance or rejection of food (Kabir et al. 2024). Synthetic dyes are more commonly used in the food processing sector than natural dyes because of their lower cost and improved stability during processing and preservation. However, synthetic dyes have been linked to allergic reactions and adverse health effects, prompting criticism and requiring strict regulations for their use (Islam, Repon, et al. 2023). As a result, natural dyes have garnered more attention than synthetic dyes. In addition to their coloring effects, natural colors provide an additional significant advantage because of their rich content of bioactive compounds. These bioactive compounds, including polyphenols and carotenoids, have various health benefits with promising antioxidant and anti‐inflammatory properties (Hasan, Islam, kabir, et al. 2024; Hasan, Islam, Haque, et al. 2024). For example, anthocyanins found in natural red and purple dyes have been linked to cardiovascular health and improved cognitive function (Olas et al. 2021; Hasan et al. 2022). Therefore, the shift toward natural colors not only addresses concerns about synthetic dyes; it also aligns with growing consumer preferences for products that offer potential health advantages beyond mere visual appeal.

Betalains are water‐soluble alkaloid substances that have evolved into biocompatible, nonhazardous pigments, antioxidants, anti‐inflammatory agents, and natural substitutes for the plant kingdom's widespread anthocyanins (Thivya et al. 2023; Abdo et al. 2023). They can also be regarded as alternatives when applied as coloring substances in the food industry (Calva‐Estrada et al. 2022). Recent studies have demonstrated that betalains exhibit approximately twice the free radical scavenging activity of anthocyanins, as assessed by ABTS+ and TEAC assays (Fernando et al. 2021; Fu et al. 2020). Furthermore, betalains have garnered significant attention over anthocyanins because of their superior bioavailability to other flavonoids and outstanding stability across a wide pH range (3–7) (Abdo et al. 2023; Dhiman et al. 2021). Betalains can be found in various plant parts, such as flowers, fruits, roots, leaves, stalks, seeds, and grains. They are derived from a wide range of species, although the majority are not cultivated for food. *Basella alba* L., often well‐known as malabar spinach, is found in tropical and subtropical climates and bears dark purple fruits. Limited studies have investigated the presence of betalain group pigments in this species (Sagar et al. 2022). To demonstrate their functional qualities, MSFEs rich in betalains were found to exhibit both cytotoxicity and antioxidant activity against human cervical carcinoma cells (Sutor‐Świeży et al. 2024). The stability of these betalain pigments, which is dependent on manufacturing and storage conditions such as pH, temperature, and water activity, among other factors, is gaining greater attention (Calva‐Estrada et al. 2022).

Extraction methods with associated factors affecting the stability of betalains via conventional and nonconventional techniques are crucial for optimizing efficiency and product quality. Prolonged extraction times are directly associated with increased energy consumption during operation, making conventional methods occasionally regarded as impractical. As a result, the drawbacks of traditional approaches have been offset by nonconventional extraction techniques. Ultrasound enhances the extraction efficiency of natural pigments by eliminating organic solvents, operating at low temperatures, and reducing the extraction time (Linares and Rojas 2022; Alam, Akther, et al. 2023). This method has gained popularity for extracting natural pigments because it creates cavitation, which ruptures the cell walls of the solid matrix, allowing for enhanced mass transfer and solvent penetration, thus promoting the extraction process. Moreover, betalains have become increasingly popular as natural pigments and are used to color a variety of foods, such as yogurt (Schneider‐Teixeira et al. 2022), ice cream (Gengatharan et al. 2021), and jelly (Nabi et al. 2023).

Yogurt is a globally consumed fermented dairy product valued for its favorable health benefits and sensorial attributes (Abdo et al. 2023). Studies have associated yogurt intake with the prevention of osteoporosis, cardiovascular disease, and hyperglycemia, as well as the mitigation of chronic diseases such as diabetes and renal disorders (Atwaa et al. 2024; Stuivenberg et al. 2023). In addition to its nutritional value, the visual appeal of yogurt, particularly its color, plays a pivotal role in shaping consumer preferences. The incorporation of fruit extracts can not only enhance the color but also increase the nutritional profile of yogurt (Ye et al. 2022). Several studies have examined the potential of incorporating plant‐based extracts into yogurt to enhance its nutritional profile and sensory qualities. For example, Guneser (2021) investigated the stability of betalains and associated color changes, whereas Ahmed et al. (2025) examined the quality parameters and shelf life of yogurt containing beetroot extract as a natural source of betalain. In a similar study, Navarro et al. (2022) explored the potential of *Opuntia robusta* , a promising alternative source of betalains, as a natural coloring agent in yogurt. Therefore, these studies underscore the potential of plant‐based additives to increase betalain concentrations, thereby contributing to the improved quality and functional properties of yogurt. Since the visual aspects of yogurt strongly influence consumer choices both during purchase and consumption (Sigwela et al. 2022), the use of natural colorants is increasingly favored over synthetic alternatives, which may have adverse health effects. However, ultrasonic‐aided MSFE has not been widely employed in research for extracting pigments and applying them to food products, particularly yogurt. Therefore, the purpose of this study was to incorporate MSFE as a natural food color in yogurt and evaluate its impact on physicochemical characteristics, color stability, bioactive and antioxidant activity, and bacterial viability during 21 days of storage at 4°C.

## 2 Materials and Methods

Malabar spinach ( *Basella alba* L.) fruit, traditional yogurt as a starter culture, sugar, and fresh cow milk were acquired from the local market (23°13′26″ N and 89°9′50″ E) of the Churamonkati Union, Jashore, Bangladesh. The fruits were harvested at peak maturity and selected on the basis of their uniform size, shape, and dark purple color, ensuring that they were disease‐free. The fruits were cleaned with distilled water and air‐dried at room temperature before further processing.

### 2.1 Chemicals and Reagents

The following items were acquired from Sigma Aldrich (St. Louis, MO, USA): quercetin (product code: Q4951, purity: ≥ 95%), sodium carbonate (Na_2_CO_3_), gallic acid, sodium hydroxide, hydrochloric acid (product code: 320931, purity: 37%), aluminum trichloride (product code: 294713), sodium acetate (product code: 241245), 2,2‐diphenyl‐1‐picrylhydrazyl (DPPH, product code: D9132‐1G), Folin–Ciocalteu reagent (FCR, product code: F9252), ethanol (EtOH, purity: 99.8%) and nutrient agar (product code: 70148).

### 2.2 Preparation of Malabar Spinach Fruit Extract (MSFE)

The method outlined by Lazăr et al. (2022), with slight modifications, was used to lyophilize malabar spinach fruit via the Manifold Freeze Dryer LBFD‐D11 (Labtron, UK). The freeze‐dried fruit powder sample was obtained by grinding (HMG‐550 W, Japan, India) and sieving (mesh size 80 μm). For further analysis, the resulting powder was placed in a low‐density polyethylene bag and kept at −18°C.

The extraction was carried out with water as the extraction solvent. First, 1 g of finely ground malabar spinach fruit powder was combined with 25 mL of water and then subjected to extraction via an ultrasonic water bath (Elma, Germany, 44 kHz) for 30 min at 50°C (Fernando et al. 2021). After extraction, the extract was allowed to cool to room temperature. It was then filtered through Whatman No. 1 filter paper in a dark atmosphere and immediately added to the yogurt to minimize its quality loss.

### 2.3 Preparation of MSFE Yogurt

**FIGURE 1:**
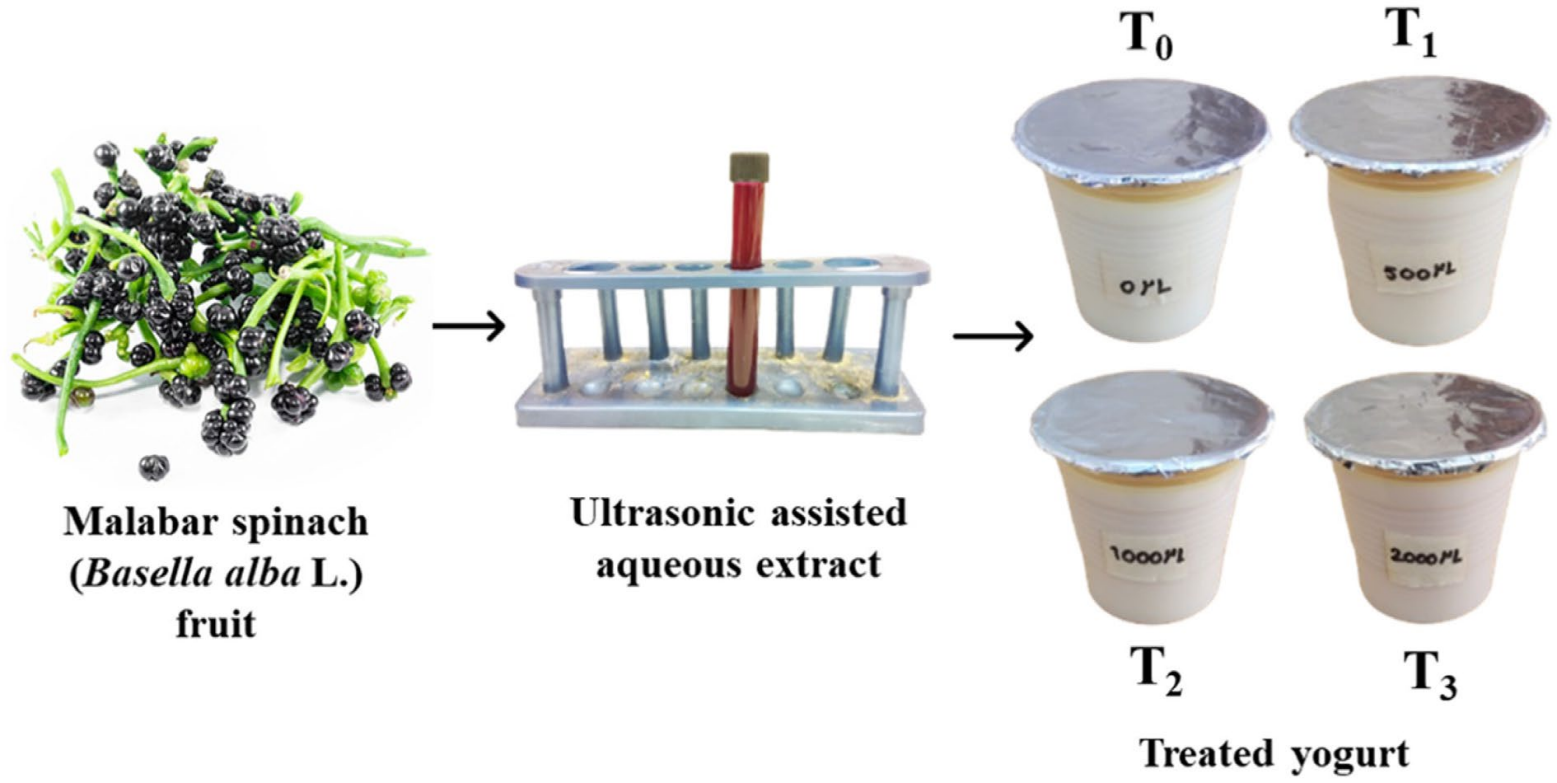
Yogurt formulations with different concentrations of MSFE.

In accordance with the methods of Bchir et al. (2020), yogurt was prepared with minor changes. First, the milk was heated to 90°C for 30 min. After pasteurization, 8% (w/v) sugar was added to the milk before it cooled to 42°C. Then, 3% (w/v) traditional yogurt was inoculated into the milk as a starter culture (El‐Messery et al. 2021). The mixture was separated into four equal amounts of 100 mL polyethylene terephthalate (PET) cups. Then, the ultrasound‐extracted MSFE was added to the milk at different concentrations: 0 μL (T_0_), 500 μL (T_1_), 1000 μL (T_2_), and 2000 μL (T_3_), followed by the treatment protocol of Schneider‐Teixeira et al. (2022) with minor modifications (Figure 1). The samples were incubated at 40°C for approximately 6 h, leading to coagulation and a final pH of 4.25 ± 0.02. A total of 152 individual yogurt cups were stored under refrigerated conditions at 4°C for subsequent quality assessment. The samples were covered with aluminum foil to minimize light exposure and limit oxygen permeability and were stored in the dark without being opened until analysis to preserve their original condition throughout the storage period.

### 2.4 Physicochemical Properties of MSFE Yogurt

#### 2.4.1 pH and Titratable Acidity

A benchtop pH meter (HI 2211, Hanna Instruments, Romania) was used to measure the pH of the yogurt samples after it was calibrated with pH 7.0 and pH 4.0 buffer solutions (Alam, Biswas, et al. 2023). The titratable acidity (%) was determined via titration with 0.1 N NaOH (El‐Messery et al. 2021), and the amount of lactic acid was calculated via Equation (1):

(1) $$\text{Titratable Acidity}\left(\%\right)=\frac{\text{Titre value}\times 0.1\;N\times 0.09008}{\text{Volume of sample}}\times 100$$

#### 2.4.2 Viscosity and Syneresis

The viscosity of the yogurt samples was measured via a rotational viscometer (Brookfield Viscometers Ltd., Harlow, UK) at a speed of 60 rpm for 40 s via Spindle No. 04 (Kim et al. 2020) and expressed as mPa.s. The method outlined by Flores‐Mancha, Ruíz‐Gutiérrez, Rentería‐Monterrubio, et al. (2021) was used to determine the degree of syneresis. In brief, 5 g of yogurt was centrifuged (DSC‐200 T, Digisystem, Taiwan) at room temperature at 2400 × *g* for 10 min. The supernatant was weighed and calculated as follows:

(2) $$\text{Syneresis}\left(\%\right)=\frac{\text{Weight of supernatant}\;\left(g\right)\;}{\text{Weight of sample}\;\left(g\right)}\times 100$$

#### 2.4.3 Total Soluble Solids (TSSs)

A digital refractometer (HI 96801, Hanna Instruments, Romania) was used to measure the TSS content of the yogurt samples, and the results are presented as %Brix.

#### 2.4.4 Color Measurements

The color parameters of the yogurt samples were measured via a colorimeter (BCM‐110, Biobase, China). The CIELAB color space, consisting of L* (lightness), a* (redness/greenness), and b* (yellowness/blueness), was employed to quantify the perceived color attributes of the yogurt. The total color change (∆E) was also determined via Equation (3):

(3) $$∆E=\sqrt{{\left(L_{0}^{*}-L^{*}\right)}^{2}+{\left(a_{0}^{*}-a^{*}\right)}^{2}+{\left(b_{0}^{*}-b^{*}\right)}^{2}}$$

where, $L_{0}^{*}$, $a_{0}^{*}$, and $b_{0}^{*}$ are the color values of the control yogurt and $L^{*}$, $a^{*}$, and $b^{*}$ are the color values of the treated samples. To ensure accurate color measurements, the colorimeter was calibrated with deionized water with a known color value of L* = 100, a* = 0, and b* = 0 (Alam, Biswas, et al. 2023).

### 2.5 Bioactive Compounds of MSFE Yogurt

#### 2.5.1 Preparation of Yogurt Extract

The extract from yogurt samples was prepared following the method described by Jouki et al. (2021) with minor modifications. In brief, 1 g of yogurt was combined with 9 mL of EtOH and vortexed (MS‐M200V Vortex Mixer, China) for 15 min to facilitate extraction. The mixture was filtered through Whatman No. 1 filter paper after being centrifuged for 15 min at 1896 × *g*. The resulting extracts were stored at −18°C for future analysis.

#### 2.5.2 Total Phenolic Content (TPC) and Total Flavonoid Content (TFC)

The TPC of MSFE‐incorporated yogurt was measured according to the protocol described by Hasan et al. (2022). Briefly, 0.25 mL of yogurt extract was mixed with 0.25 mL of FCR, and then 0.5 mL of 7.5% (w/v) Na_2_CO_3_ solution was added. The final volume of 5 mL was adjusted with distilled water and vortexed for 10 s. Then the mixture was centrifuged at 1896 × *g* for 15 min and allowed to develop color for 40 min at room temperature in the dark. Finally, the absorbance of the sample was measured at 765 nm via a UV–visible spectrophotometer (T60, PG Instruments Ltd., UK). Using gallic acid as a standard, the data are reported as mg gallic acid equivalents per 100 g of dry matter (mg GAE/100 g DM).

The TFC of the MSFE yogurt was assessed via the modified colorimetric method of AlCl_3_ according to the protocol of Shraim et al. (2021). In brief, 0.5 mL of yogurt extract was mixed with 2 mL of EtOH and 0.20 mL of 10% (w/v) AlCl_3_. After vortexing, the mixture was allowed to rest for 5 min. Next, 0.20 mL of CH_3_COONa (1.80 g/mL) was added, followed by the addition of EtOH to bring the final volume to 5 mL. The mixture was incubated in the dark at room temperature for 40 min to allow color development. The absorbance of each sample was subsequently measured at 430 nm. Using quercetin as a standard, the results were calculated and expressed as mg of quercetin equivalents per 100 g of dry matter (mg QE/100 g DM).

#### 2.5.3 DPPH Free Radical Scavenging Assay

The free radical scavenging ability of MSFE‐containing yogurt was assessed via a modified version of the method described by Kabir et al. (2024). First, 4 mg of DPPH was dissolved in 100 mL of EtOH to create an ethanolic DPPH solution (0.1 mM), which was then allowed to stand overnight to fully dissolve. Subsequently, 50 μL of yogurt extract was combined with 1.95 mL of DPPH solution, vortexed, and then incubated in the dark at room temperature for 30 min. The absorbance of the mixture was measured at 517 nm, and the scavenging capacity (% inhibition) was calculated via Equation (4).

(4) $$\text{DPPH}\;\left(\%\;\text{inhibition}\right)=\frac{\text{Absorbance}\;\left(\text{blank}\right)-\text{Absorbance}\;\left(\text{sample}\right)}{\text{Absorbance}\;\left(\text{blank}\right)}\times 100$$

### 2.6 Total Betalain Content (TBC)

The TBC of the MSFE yogurt was determined according to Basavaraja et al. (2022). The absorbance was measured at 535 nm and 480 nm for betacyanin (BC) and betaxanthin (BX) respectively. The BC, BX, and TBC were calculated via the following equations.

(5) $$\mathrm{BC}\;\left(\mathrm{mg}/L\right)=\frac{\text{Absorbance}\;\mathrm{at}\;535\;\mathrm{nm}\times \text{dilution factor}\times \text{molecular weight}\;\left(550\;\frac{g}{\mathrm{mol}}\right)\times 100}{\text{Extinction factor}\;\left(60,000\;\frac{L}{\mathrm{mol}\;\mathrm{cm}}\right)\times \text{width of cuvette cell}\;\left(1\;\mathrm{cm}\right)}$$

(6) $$\mathrm{BX}\;\left(\mathrm{mg}/L\right)=\frac{\text{Absorbance}\;\mathrm{at}\;480\;\mathrm{nm}\times \text{dilution factor}\times \text{molecular weight}\;\left(308\;\frac{g}{\mathrm{mol}}\right)\times 100}{\text{Extinction factor}\;\left(48,000\;\frac{L}{\mathrm{mol}\;\mathrm{cm}}\right)\times \text{width of cuvette cell}\;\left(1\;\mathrm{cm}\right)}$$

(7) $$\mathrm{TBC}\;\left(\mathrm{mg}/L\right)=\mathrm{BC}\;\left(\mathrm{mg}/L\right)+\mathrm{BX}\;\left(\mathrm{mg}/L\right)$$

### 2.7 Color Stability of MSFE Along the pH Scale

The effect of pH on the qualitative color stability of MSFEs was determined via the use of 13 vials, according to Schneider‐Teixeira et al. (2022) with minor changes. Briefly, vial 1 was filled with 5 mL of HCl (0.1 mol/L, estimated pH 1), vial 13 was filled with 5 mL of NaOH (0.12 mol/L, estimated pH 13), and the rest of the vials (vials 2–12) were filled with 4.5 mL of distilled water (neutral pH). A 0.5 mL solution from vial 1 was placed in vial 2 (estimated pH 2), followed by adequate mixing. Then, 0.5 mL of solution from vial 2 was placed in vial 3 (with an estimated pH of 3), and the process continued up to vial 6 to make a series of dilutions. A similar process was followed for successive dilutions of the basic solution (vial 13 to vial 8). Vial 7 was left with only distilled water (estimated pH 7). Finally, each vial was filled with 0.5 mL of previously prepared MSFE. The color stability of the MSFEs, along with the pH scale, was observed during 21 days of refrigerated storage.

### 2.8 Total Plate Count

The total aerobic bacteria were enumerated according to Cho, Kim, et al. (2020) with some modifications. First, 0.1 g of yogurt sample was serially diluted with 0.9 mL of sterile distilled water. The diluted mixture was subsequently vortexed thoroughly, and 0.1 mL of each diluted sample was spread onto nutrient agar plates. The plates were then incubated at 37°C for 24 h. The viable bacterial populations were expressed as CFU/g.

### 2.9 Sensory Evaluation of MSFE Yogurt

The sensory evaluation was conducted by 20 semi‐trained panelists comprising the faculty members of the Department of Food Engineering of Jashore University of Science and Technology, Jashore, Bangladesh. The Ethical Review Committee of the Faculty of Biological Science, Jashore University of Science and Technology, Jashore, Bangladesh (ERC/FBST/JUST/2024–202), approved all relevant rules, guidelines, and regulations followed in this study. The participants were thoroughly informed about the study's objectives, procedures, and potential risks of allergens. The yogurt samples were given to the participants in PET cups labeled with a unique three‐digit code and served at room temperature. They were encouraged to drink mineral water between each sample to avoid aftertaste (Islam, Biswas, et al. 2023). Each panelist received a blind sensory evaluation sheet and was asked to rate the yogurt's appearance, smell, color, taste, texture, and overall acceptability on a 9‐point hedonic scale, where 1 indicated strongly dislike and 9 indicated strongly like (Kabir et al. 2024). An open‐ended questionnaire session was conducted among participants to gather their subjective opinions about yogurt.

### 2.10 Statistical Analysis

The experiments were conducted in a completely randomized design (CRD) in a 4 × 4 factorial scheme. All trials were performed in triplicate. The data were analyzed via IBM SPSS Statistics 23.0 software (SPSS Inc., USA) and are reported as the means ± standard deviations. Tukey's honest significant difference test (*p* < 0.05) was employed to compare means between treatment groups. The Pearson correlation between each antioxidant property and the principal component analysis (PCA) of the sensory data was performed via Origin software (version 10.1.0.170, OriginLab, USA).

## 3 Results and Discussion

### 3.1 Physicochemical Properties of MSFE Yogurt

#### 3.1.1 pH and Titratable Acidity (TA)

**FIGURE 2:**
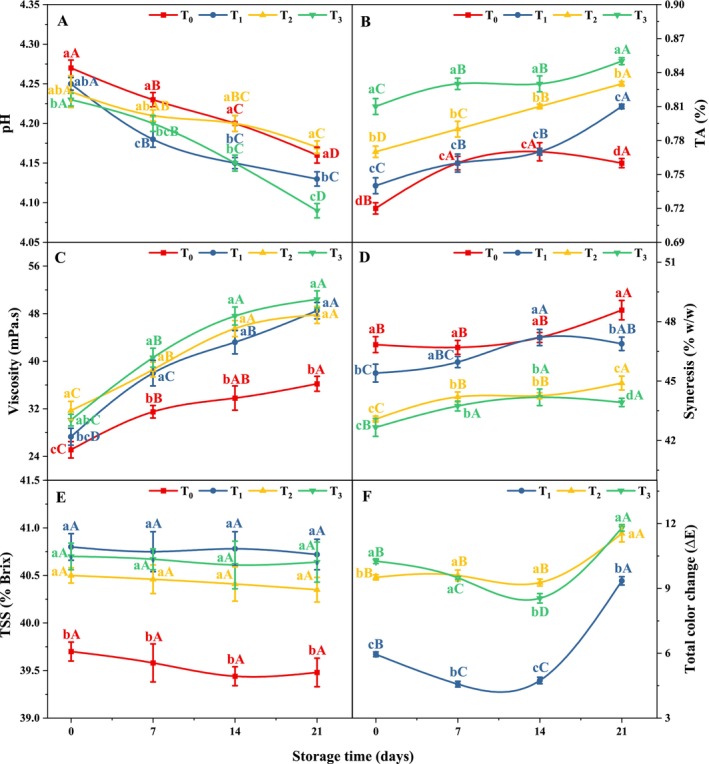
Effects of treatment and storage on (A) pH, (B) TA, (C) viscosity, (D) syneresis, (E) TSS, and (F) total color change (ΔE) in MSFE‐containing yogurt. Statistical differences between treatments within the same storage day (*p* < 0.05) are indicated by distinct lowercase letters (a–d), and significant differences between storage days within the same treatment (*p* < 0.05) are indicated by distinct uppercase letters (A–D). T_0_ = 0 μL, T_1_ = 500 μL, T_2_ = 1000 μL, and T_3_ = 2000 μL of MSFE added per 100 mL of yogurt.

pH and TA are important factors used to verify the quality of yogurt. The changes in the pH and TA of yogurt during storage are depicted in Figure 2A,B, respectively. With increasing MSFE concentration in yogurt, the pH decreased (*p* < 0.05), whereas the titratable acidity increased (*p* < 0.05). The MSFE‐formulated yogurt presented a lower pH ranging from 4.27–4.23 and a greater acidity between 0.72% and 0.81%. The TA results indicate that all the yogurt samples met the Codex Alimentarius minimum titratable acidity requirement of 0.6% (Adjei et al. 2024). The acidic conditions of MSFE‐containing yogurt can be explained by the lower pH value of MSFE, which varies between 5.14 and 5.20 (Nur, Khan, et al. 2023). Halladj et al. (2022) reported a reduction in pH and promotion of TA with the inclusion of beetroot cooking water in yogurt in a dose‐dependent manner. However, after 21 days of storage, a significant reduction (*p* < 0.05) in pH and an increase (*p* < 0.05) in TA were observed in the yogurt samples. The values of pH and TA varied between 4.09 and 4.16 and between 0.76% and 0.85%, respectively, at the end of storage. After 21 days, the pH decreased by 2.6%, 2.82%, 1.65%, and 3.3%, whereas the TA increased by 5.6%, 9.5%, 7.8%, and 4.9% in T_0_, T_1_, T_2_, and T_3_, respectively, compared with the initial storage. The acid‐tolerant characteristics and high metabolic activity of lactic acid bacteria such as *Lactobacillus bulgaricus* cause post acidification due to rapid lactic acid production, thus increasing the TA content and decreasing the pH in yogurt (Kabir et al. 2021; Wijesekara et al. 2022). Abdo et al. (2023) reported comparable outcomes, observing a decrease in the pH and an increase in the acidity of yogurt toward the end of storage.

#### 3.1.2 Viscosity and Syneresis

As shown in Figure 2C,D, MSFE increased (*p* < 0.05) the viscosity of yogurt from 25.1 (T_0_) to 30.1 mPa.s (T_3_), and with this increase in viscosity, syneresis decreased (*p* < 0.05) from 46.84% (T_0_) to 42.66% (T_3_) at the initial stage. Kabir et al. (2021) reported that including dietary fiber in yogurt might increase viscosity. The addition of MSFE may have increased yogurt viscosity, as *Basella alba* L. fruit is an ample source of dietary fiber (29.47–34.58 g/100 g) (Nur, Khan, et al. 2023). Furthermore, the increase in MSFE concentration in yogurt led to increased water‐holding capacity and decreased syneresis because a higher concentration of fiber present in MSFE can bind with water during the initial storage of yogurt (Al‐Sahlany et al. 2023). Similar findings were also reported by Cho, Hwa, et al. (2020), who reported that adding 0.5% sugared omija extract decreased syneresis and increased the viscosity of yogurt. Following a 21‐day storage period, the yogurt samples presented a substantial increase (*p* < 0.05) in viscosity and syneresis. At the final storage conditions, the yogurt viscosity ranged from 36.2 (T_0_) to 50.4 mPa.s (T_3_), and their syneresis was between 48.58% (T_0_) and 43.92% (T_3_). The viscosity increased by 44.2%, 77.7%, 50.8%, and 67.4%, while syneresis increased by 3.7%, 3.3%, 4.2%, and 3% in comparison to the first day of storage in T_0_, T_1_, T_2_, and T_3_, respectively. Increased acidity may lead to a more compact protein matrix (Abdo et al. 2023), and enhanced milk coagulation and structural recovery during yogurt storage (Basiony et al. 2023; Kabir et al. 2021), which may contribute to increased viscosity, supporting these findings.

#### 3.1.3 Total Soluble Solids (TSSs)

Figure 2E shows a significant dose‐dependent increase (*p* < 0.05) in the TSS content upon the addition of MSFE to yogurt, followed by a gradual nonsignificant decrease (*p* > 0.05) in the TSS content over time. Initially, the TSS content of the MSFE yogurt ranged from 39.7% (T_0_) to 40.7% Brix (T_3_). The low but considerable amount of total sugar present (6.13%–7.45% Brix) in MSFE could be the reason for this variation in TSS in yogurt (Nur, Khan, et al. 2023). These results agreed with previous reports in aloe vera jell yogurt (Ikram et al. 2021) and black rice beverage yogurt drink (Atwaa et al. 2024), which similarly revealed a dose‐dependent increase in the TSS content upon extract addition. All the yogurt samples presented a minor reduction (*p* > 0.05) in the TSS content throughout the 21‐day storage period. As expected, the maximum TSS (40.64% Brix) was observed in T_3_, and the minimum TSS (39.48% Brix) was observed in T_0_. By the end of the storage period, the TSS content had decreased by 0.6 (T_0_), 0.2 (T_1_), 0.4 (T_2_), and 0.15% (T_3_) in the yogurt samples compared with the initial storage day. A study by Ikram et al. (2021) suggested that the decrease in TSS during storage could be attributed to increased microbial activity triggered by the presence of sugars. However, more whey separation or syneresis results in a lower TSS content. These results are consistent with those of Almusallam et al. (2021), who reported a slight decrease in the total solid content of date palm spikelet extract incorporated in yogurt over the storage period.

#### 3.1.4 Color Parameters

Color is a critical attribute of fruit yogurt, affecting its sensory appeal, consumer preference, and overall shelf stability. The total color change (∆E) shown in Figure 2F is important because all differences in L*, a*, and b* color values between the MSFE yogurt and the control yogurt were considered. Initially, the color of the control yogurt was pale yellow with the L* (lightness) and b* (yellowness) values of 89.4 and 17.53, respectively. MSFE significantly decreased (*p* < 0.05) the lightness and yellowness of yogurt, from 85.6 to 82.8 and 13.1 to 11.26, respectively. With decreasing L* and b* values, redness (a*) increased significantly (*p* < 0.05) in the yogurt samples with the addition of MSFEs ranging from −1.76 to 2.97, which also influenced the visual appearance of the yogurt. As predicted, betalain present in MSFE reduced the lightness and yellowness as well as increased redness in yogurt, which was also correlated with the noticeable color change (∆E) in the treated samples (5.95–10.26) compared with the control. These results were consistent with those of Rocha et al. (2022) and Abdo et al. (2023) who reported similar changes in color parameters in yogurt formulated with beetroot syrup and beetroot stalk extract, respectively. A substantial increase in lightness and yellowness was observed in the yogurt samples after 14 days of storage. After the second week of storage, the L* and b* values were 92.8–85.6 and 21.45–19.21, respectively. On the other hand, redness (a*) decreased in the MSFE yogurt, varying between −1.9 and 2.1. The maximum change in color (∆E) in yogurt after two weeks was pronounced at T_2_ (9.27), whereas the other samples had ∆E values of 4.74 (T_1_) and 8.54 (T_3_). The observed increase in L* and b* with a significant decrease in the a* value likely resulted from the degradation of betalain within the yogurt matrix. Hydrolysis or isomerization reactions in yogurt may have led to the formation of isobetanin, isobetanidin, and neobetanin, indicating that betalain degradation contributed to the observed shifts in color parameters (Sadowska‐Bartosz and Bartosz 2021).

At the end of storage, the redness (a*) slightly decreased, whereas the lightness and yellowness slightly increased compared with those on the initial day of storage. After 21 days, the lightness, redness, and yellowness ranged from 93.4 to 83.5, −1.92 to 2.49, and 21.25 to 16.59, respectively, which also supported the total color change (9.35–11.8). Similar findings were also reported by Abdo et al. (2023) and Flores‐Mancha, Ruíz‐Gutiérrez, Sánchez‐Vega, et al. (2021) with the addition of beetroot stalk extract and beetroot juice, respectively. According to their findings, the subsequent decreases after the second week, along with the increase in redness (a*) after 21 days, could be attributed to the regenerative ability of betalain and microbial activity during storage, which caused an intense release of betalain in yogurt.

### 3.2 Bioactive Compounds of MSFE Yogurt

#### 3.2.1 Total Phenolic Content (TPC) and Total Flavonoid Content (TFC)

**FIGURE 3:**
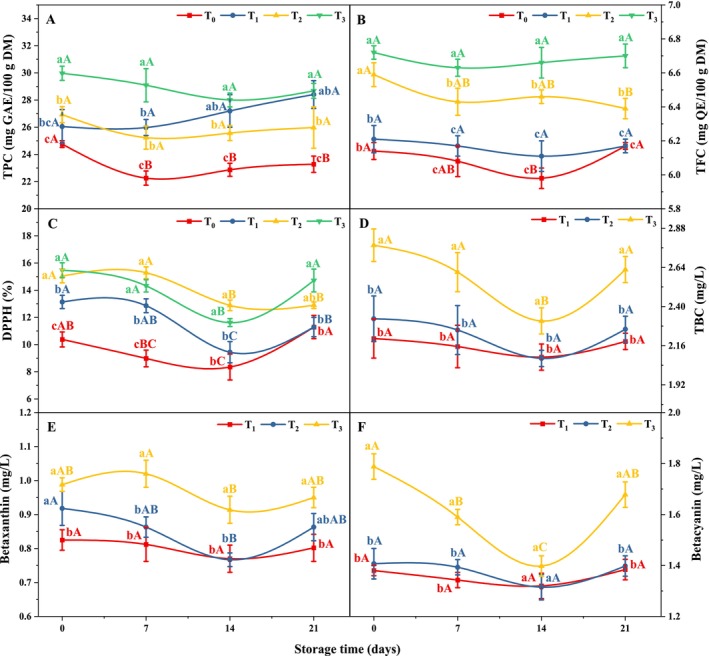
Effects of treatment and storage on (A) TPC, (B) TFC, (C) DPPH, (D) betalain, (E) betaxanthin, and (F) betacyanin of MSFE yogurt. Statistical differences between treatments within the same storage day (*p* < 0.05) are indicated by distinct lowercase letters (a–d), and significant differences between storage days within the same treatment (*p* < 0.05) are indicated by distinct uppercase letters (A–D). T_0_ = 0 μL, T_1_ = 500 μL, T_2_ = 1000 μL, and T_3_ = 2000 μL of MSFE added per 100 mL of yogurt.

As shown in Figure 3A,B, the TPC and TFC of yogurt significantly increased (*p* < 0.05) with the addition of MSFEs. Under the initial conditions, MSFE increased the TPC and TFC of yogurt from 24.76 to 29.97 mg GAE/100 g DM and from 6.14 to 6.72 mg QE/100 g DM, respectively. This increase in TPC and TFC reflects the abundance of total polyphenols (125.32–181.99 mg GAE/g) and flavonoids (110.25–148.70 mg QE/g) in malabar spinach fruit (Nur, Islam, et al. 2023). A study by Ahmed et al. (2022) revealed a similar pattern of increase in TPC and TFC in yogurt supplemented with apple pomace and pomegranate peel powder. As the storage period progressed, a subsequent decrease in the TPC and TFC was observed until the second week, after which the values stabilized starting from the third week. Anuyahong et al. (2020) also reported fluctuations in the fluctuation of phytochemicals during the storage of riceberry rice extract in yogurt. After 21 days of storage, a slight decrease (*p* > 0.05) in the TPC and TFC of yogurt was noted, ranging from 23.28 to 28.68 mg GAE/100 g DM and from 6.17 to 6.70 mg QE/100 g DM, respectively. The maximum reductions in the TPC and TFC were observed in T_0_ (6%) and T_2_ (3.03%), whereas the minimum reductions were observed in T_1_ (3.52%) and T_3_ (0.3%), respectively. These findings concur with those of a previous study of black rice‐enriched yogurt by Atwaa et al. (2024). The reduction in bioactive compounds in yogurt may be attributed to several factors, including the decreased pH resulting from increased metabolic activity of lactic acid bacteria and an extended fermentation period. Additionally, the formation of protein‐polyphenol complexes in milk could enhance the masking effect of phenolic compounds (Kabir et al. 2021; Saberi et al. 2023).

#### 3.2.2 DPPH Free Radical Scavenging Activity

The addition of MSFE had a significant influence on the antioxidant activity of yogurt, as indicated in Figure 3C. The percent inhibition of all the samples significantly improved (*p* < 0.05) with increasing MSFE concentration. The MSFE initially exhibited significant free radical scavenging activity (*p* < 0.05), ranging from 10.38% (T_0_) to 15.47% (T_3_) inhibition in the yogurt samples. The higher antioxidative capacity (IC_50_ values of 21.55 to 34.37 μg/mL) of MSFEs could be the reason for the increase in percentage inhibition in MSFE‐containing yogurt (Nur, Islam, et al. 2023). Furthermore, the presence of higher levels of bioactive compounds such as phenolics and flavonoids within MSFE could contribute to the observed increase in the radical scavenging activity of yogurt (Hasan et al. 2022; Kabir et al. 2024). In alignment with the present findings, the incorporation of cactus pear juice powder (Lugo‐Zarate et al. 2021) and blue pea flower extract (Sutakwa et al. 2021) into yogurt samples resulted in comparable changes in radical scavenging activity. Similarly, studies by Kabir et al. (2024) and Kabir et al. (2021) also demonstrated that higher levels of TPC and TFC were associated with increased DPPH free radical scavenging activity in mango peel powder‐incorporated noodles and banana peel extract yogurt, respectively. A notable decrease (*p* < 0.05) in % inhibition in all the samples was observed until the second week of storage, after which the value later recovered after 21 days. Anuyahong et al. (2020) reported an increase and subsequent decrease in the DPPH radical scavenging activity of riceberry extract yogurt, while Flores‐Mancha, Ruíz‐Gutiérrez, Sánchez‐Vega, et al. (2021) noted a decrease and subsequent increase in free radical scavenging activity during storage. After storage, T_3_ resulted in the highest retention of free radical scavenging activity (4.8% decrease), whereas T_2_ resulted in the lowest retention (14.2% decrease).

The observed decrease in antioxidant activity in fortified yogurt during storage may be attributed to the formation of protein‐polyphenol complexes in milk (Saberi et al. 2023). Casein, the primary milk protein, has a high affinity for polyphenolic compounds because of the availability of numerous binding sites, facilitating strong noncovalent interactions such as hydrogen bonding and hydrophobic interactions. These interactions can lead to the structural modification of polyphenols by masking their hydroxyl groups, thereby reducing their ability to act as free radical scavengers (Kabir et al. 2021; Ahmed et al. 2025; Yildirim‐Elikoglu and Erdem 2018). This phenomenon has been widely reported in dairy‐based functional foods and highlights the importance of considering matrix effects in the design and evaluation of fortified products.

#### 3.2.3 Pearson Correlation Analysis of TPC, TFC, and DPPH in MSFE Yogurt

**FIGURE 4:**
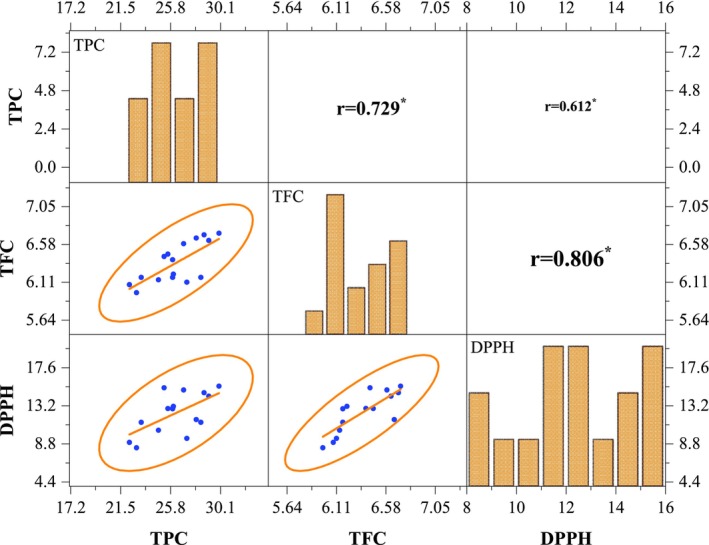
Correlations matrix between different phytochemicals (TPC, TFC, and DPPH) of MSFE‐incorporated yogurt. The lower end of the diagonal represents the scatter plot with a regression line and confidence ellipse, and the upper end represents the correlation coefficients with a significance level of *p* < 0.05 (denoted by stars). Histograms were included on the main diagonal to illustrate the distribution of each variable.

Figure 4 shows a positive correlation between the TPC, TFC, and DPPH radical scavenging activity in the MSFE yogurt samples. These results suggest a potential collaborative effect among these phytochemicals in contributing to the antioxidant capacity of yogurt. A significant correlation was considered to exist between two distinct parameters when their absolute Pearson correlation coefficient exceeded 0.75, indicating a positive or negative association between those parameters (Boonupara et al. 2024; Hasan, Islam, kabir, et al. 2024).

TFC was strongly positively correlated (*r* = 0.806; *p* < 0.05) with DPPH. Flavonoids possess a notable ability to scavenge reactive oxygen species, thereby demonstrating promising antioxidant activity (Muflihah et al. 2021). A significant positive correlation of TPC was observed between TFC (*r* = 0.729; *p* < 0.05) and DPPH (*r* = 0.612; *p* < 0.05) in the MSFE yogurt. These findings suggest that flavonoids are a primary contributor to the overall phenolic composition of yogurt, and that the TPC plays a crucial role in enhancing the ability of yogurt to scavenge free radicals. Almusallam et al. (2021) also reported an increase in phytochemicals by adding date palm spikelet extract to yogurt, which supports the findings of the present study.

### 3.3 Total Betalain Content (TBC)

As shown in Figure 3D, a notable increase (*p* < 0.05) in TBC was observed in MSFE‐incorporated yogurt in a concentration‐dependent manner. At the initial storage stage, the TBC ranged from 2.204 to 2.776 mg/L. Sagar et al. (2022) attributed the increase in betalain content in yogurt to the high levels of betalain (4.26–156.42 mg/100 g FW) present in the *Basella alba* L. extract. The contents of betaxanthin (Figure 3E) and betacyanin (Figure 3F) also increased (*p* < 0.05) in yogurt with increasing MSFE concentration in a similar pattern to that of betalain. During initial storage, the betaxanthin and betacyanin contents ranged from 0.825 to 0.988 mg/L and from 1.38 to 1.788 mg/L, respectively. After the second week of storage, a substantial decrease in betalain, betaxanthin, and betacyanin contents was observed among the samples. During this storage period, T_1_ decreased by 5.17%, 6.67%, and 4.35%; T_2_ decreased by 10.45%, 16.45%, and 6.54%; and T_3_ decreased by 16.71%, 7.49%, and 21.81% in betalain, betaxanthin, and betacyanin, respectively. This degradation of betacyanin may result from hydrolysis, resulting in the production of cyclo‐DOPA‐D‐glucoside (CDG) and betalamic acid. This degradation reaction is reversible (Flores‐Mancha, Ruíz‐Gutiérrez, Sánchez‐Vega, et al. 2021), as further evidenced after 21 days of storage. After the storage, the TBC increased from 2.186 to 2.627 mg/L, the betacyanin content increased from 1.384 to 1.678 mg/L, and the betaxanthin content increased from 0.802 to 0.95 mg/L. Betacyanin can be reformed through Schiff‐base condensation between the amine group of CDG and the aldehyde group of betalamic acid. Moreover, betalamic acid regenerates betaxanthin in the presence of amino acids, confirming the regenerative ability of betalain in yogurt (Abdo et al. 2023).

The regeneration of betacyanins and betaxanthins involves hydrolysis products present in yogurt, primarily through a Schiff‐base condensation reaction between the amine group of CDG and the aldehyde group of betalamic acid. Upon mixing these two compounds in solution, betalain—a derivative of both betacyanin and betaxanthin—is rapidly reformed (Abdo et al. 2023; Sadowska‐Bartosz and Bartosz 2021). A study by Flores‐Mancha, Ruíz‐Gutiérrez, Sánchez‐Vega, et al. (2021) and Ahmed et al. (2025) similarly reported patterns of betalain degradation and resynthesis in yogurt formulated with beetroot extract. They highlighted the role of the Schiff‐base condensation reaction between CDG and betalamic acid, which supports the findings of the present study.

### 3.4 Color Stability of Betalain in MSFEs

**FIGURE 5:**
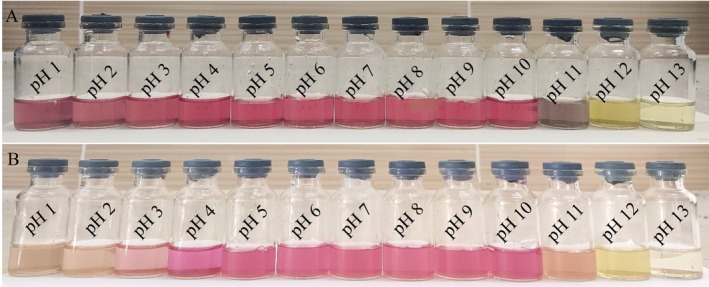
Color stability of MSFE at different pH values; (A) Day 0 and (B) day 21 of storage.

Betalain is a color‐stable pigment with a wide range of pH values (Guneser 2021). The color stability of the MSFEs is demonstrated in Figure 5A,B, which depict the pH range from 1 to 13 on day 0 and day 21, respectively, under 4°C storage conditions. As shown in Figure 5A, the vibrant red color of the extract did not significantly change at pH 3–10, which is in the range of MSFE‐incorporated yogurt pH (4–5). According to Fu et al. (2020), betalain is optimally stable at pH 4–6, which is in agreement with the present study. Additionally, the extract lost its characteristic red color at pH ≤ 3 and pH ≥ 11; thus, its betalain became unstable in this pH range. Skalicky et al. (2020) suggested that structural changes in betalain molecules could occur under both acidic (pH < 7) and alkaline (pH > 7) conditions. Acidic conditions induce betalain conversion to a cationic form, resulting in a violet color. Conversely, alkaline conditions lead to aldimine bond hydrolysis, yielding betalamic acid and CDG, and a yellow‐brown color change (Miguel 2018). Nur, Khan, et al. (2023) reported that the maximum color retention of MSFEs occurs in the pH range of 4–6. Similarly, Nirmal et al. (2021) reported that betalain extracted from beetroot gained maximum stability at pH 3–7.

The color degradation of *Basella alba* L. extract was strongly visualized at pH ≤ 3 and pH ≥ 11 but minimally visualized at pH 4–10 after 21 days of refrigerated storage. The slight degradation of betalain content at pH 4–10 highlights the stability of betalain in yogurt during storage, as confirmed by the spectrophotometric analysis of TBC. Dehydrogenation, decarboxylation, or hydrolytic cleavage of betacyanin may cause structural modifications, resulting in the loss of betacyanin and color degradation of the extract during storage (Schneider‐Teixeira et al. 2022). Rocha et al. (2022) reported a similar pattern of betalain degradation at pH 3–5 in beetroot extract after 21 days of storage at 4°C. However, this color degradation over a wide range of pH values refers to the degradation of betalain in yogurt after 21 days of storage. They can be stabilized by encapsulation, glycosylation, or the addition of anionic polysaccharides before their application as colorants in food systems (Schneider‐Teixeira et al. 2022; Skalicky et al. 2020). The addition of a chelating agent or a protein complex may also ensure the stability of betalain (Liliana and Oana‐Viorela 2020).

### 3.5 Total Plate Count

Table 1 displays the total plate count of yogurt formulated with varying amounts of MSFE. A dose‐dependent decrease (*p* < 0.05) in bacterial viability was observed, decreasing from 552.6 (T_0_) to 118.7 × 10^6^ CFU/g (T_3_) upon the addition of MSFE to the yogurt. This decrease in viability demonstrates the potent antibacterial properties of MSFE. Various secondary metabolites (e.g., saponins, tannins, and terpenes) and phytochemicals (e.g., β‐sitosterol and lupeol) present in *Basella alba* L. can disrupt microbial cell membranes by degrading their proteins, leading to cell inactivation or death (Chan et al. 2022; Nur, Islam, et al. 2023; Phimolsiripol et al. 2021). Adding 500 μL (T_1_) of MSFE to yogurt did not significantly (*p* > 0.05) lower bacterial viability; however, adding 1000 μL (T_2_) or 2000 μL (T_3_) significantly (*p* < 0.05) reduced bacterial viability. Despite the reduction, all yogurt samples consistently maintained viable probiotic counts above the internationally recommended threshold of 10^6^ CFU/g throughout the storage period, ensuring their functional integrity (Buniowska‐Olejnik et al. 2025; CODEX STAN 243–2003). These findings suggest that the microbial viability of yogurt was not considerably reduced by 500 μL of MSFE.

**TABLE 1:** Effects of different treatments and storage conditions on the total plate count (×10^6^ CFU/g) of MSFE‐containing yogurt.

Treatment	Storage (days)			
	0	7	14	21
T_0_	552.6 ± 20.2^aB^	596.2 ± 18.1^aAB^	625.3 ± 25.3^aA^	584.4 ± 33.4^aAB^
T_1_	520.5 ± 24.3^aA^	545.3 ± 26.4^aA^	589.4 ± 34.5^aA^	514.8 ± 29.6^aA^
T_2_	198.6 ± 28.4^bA^	259.1 ± 19.2^bAB^	287.6 ± 10.6^bA^	240.3 ± 37.4^bAB^
T_3_	118.7 ± 31.2^cC^	249.3 ± 28.6^bAB^	291.5 ± 30.4^bA^	185.6 ± 20.3^bBC^

*Note:* Statistically significant differences between treatments within the same storage day (*p* < 0.05) are denoted by distinct lowercase letters (a–d) within each column and statistically significant differences between storage days within the same treatment (*p* < 0.05) are indicated by distinct uppercase letters (A–D) within each row.

Abbreviations: T_0_ = 0 μL, T_1_ = 500 μL, T_2_ = 1000 μL, and T_3_ = 2000 μL of MSFE added per 100 mL of yogurt.

The bacterial viability increased significantly (*p* < 0.05) after the first week of storage and then stabilized after 21 days. At the end of the storage period, the viability ranged from 185.6 (T_3_) to 584.4 × 10^6^ CFU/g (T_0_). At the end of the 21‐day storage period, T_3_ presented the greatest increase of 56.36%, whereas T_0_ presented the lowest increase of 5.75%, compared with the initial day. According to investigations by Cho, Hwa, et al. (2020) and Rahmani et al. (2021), the microbiological viability of yogurt supplemented with omija and green tea extract, respectively, initially decreased and then increased again. Perhaps yogurt's relatively high acidity and nutritional deficiencies are responsible for the decrease in bacterial count (Ikram et al. 2021; Mukta et al. 2021). Another study by Dias et al. (2020) reported that the increased polyphenolic activity of yogurt might inhibit the growth of lactic acid bacteria.

### 3.6 Sensory Evaluation and Principal Component Analysis (PCA)

**FIGURE 6:**
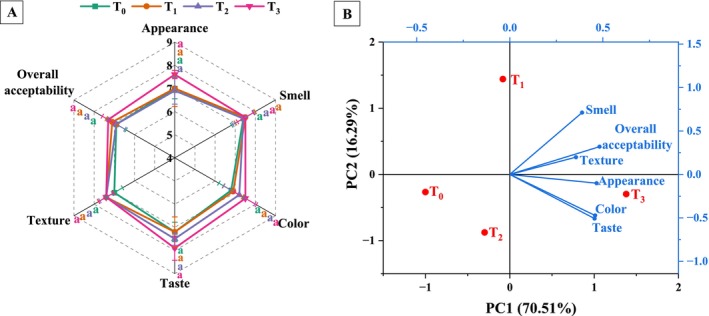
Effects of treatment and storage on (A) sensory evaluation and (B) principal component analysis (PCA) of MSFE‐incorporated yogurt. Statistical differences between treatments within the same storage day (*p* < 0.05) are indicated by distinct lowercase letters (a–d). T_0_ = 0 μL, T_1_ = 500 μL, T_2_ = 1000 μL, and T_3_ = 2000 μL of MSFE added per 100 mL of yogurt.

Figure 6A displays the effects of fortifying yogurt with varying MSFE concentrations on its sensory attributes. According to the sensory evaluation, 2000 μL of extract (T_3_) mostly increased the acceptability of yogurt compared with the other samples. Although the sensory evaluation uses a relatively small, semi‐trained sensory panel composed of individuals from the authors' institution, the panel provided meaningful insights into consumer perception. However, semi‐trained panelists may lack the precision and consistency in assessing sensory attributes that fully trained panelists possess. Moreover, the limited panel size and lack of diversity may affect the generalizability and reliability of the findings. Future studies should consider employing a larger and more diverse panel, including both trained assessors and broader consumer groups, to increase the accuracy and applicability of sensory evaluation results. Increasing the MSFE concentration non‐significantly (*p* > 0.05) improved the appearance (7.6), color (7.5), taste (7.9), and overall acceptability (7.3) of the T_3_ sample, whereas the lowest values were observed in the T_0_ sample. Panelists reported that a higher betalain content might increase the color and appearance score, confirming the instrumental analysis of the color parameters of yogurt. Nevertheless, some panelists have claimed that yogurt with relatively high levels of MSFE might present a slightly less familiar visual appearance and taste to some of its consumers, potentially influencing their acceptance. However, these visual and taste variations did not significantly affect the panelists' sensory evaluations. Similar to the present findings, beetroot peel powder increased the color score of mayonnaise because of its attractive pigment (Lazăr et al. 2022). Furthermore, higher sugar contents and color–taste synesthetes may increase the taste of MSFE‐containing yogurt (Abdo et al. 2023; Nur, Khan, et al. 2023). On the other hand, MSFE did not significantly affect the smell and textural quality of fortified yogurt (*p* > 0.05). The panelists remarked almost equally (7.4–7.5) on all the treated samples. Moreover, the control had scores of 7 and 7.4 for texture and smell, respectively, suggesting that the incorporation of MSFE did not negatively impact traditional yogurt characteristics and supported its commercial viability. Panelists claimed that the neutral smell of *Basella alba* L. could be the reason for this equality (Kozioł et al. 2024). According to the panelists' remarks, T_3_ received the highest scores across all sensory attributes, making it the most acceptable option. These findings suggest that MSFEs (up to 2000 μL) can be used as a functional coloring agent in yogurt without adversely affecting its key sensory properties. Owing to its potential antioxidant and nutritional benefits in attracting health‐conscious consumers, MSFEs present a significant commercial opportunity as a natural additive in dairy, especially within the growing functional and health‐oriented yogurt segments. Similarly, Abdo et al. (2023) reported that beetroot stalk extract can be used up to 5% as a colorant in yogurt without any negative impact.

Principal component analysis (PCA) was used to examine the relationships between different treatments and the sensory properties of MSFE‐incorporated yogurt (Figure 6B). The PCA biplot illustrates the key sensory properties of yogurt that have the most significant impact on overall acceptance after MSFEs are added to the yogurt. The results of the eigenvector loadings revealed that PC1, with an eigenvalue of 4.23, explained 70.51% of the variation, whereas PC2, with an eigenvalue of 0.98, explained 16.29% of the variation. Together, PC1 and PC2 explained 86.80% of the total variation. The PCA scores indicated that the MSFE‐incorporated yogurt was acceptable for consumption. PC1 was influenced primarily by appearance, texture, and overall acceptability, with loadings of 0.43421, 0.32413, and 0.45333, respectively. In contrast, PC2 was dominated by smell, color, and taste, with corresponding loadings of 0.66739, 0.44205, and 0.47872, respectively. The T_3_ sample was the furthest from the control sample and was closely related to positive sensory attributes, confirming that the highest dose of MSFE positively influenced the sensory properties of the yogurt compared to the control. According to the PCA results, the T_3_ sample was influenced primarily by its appearance, color, and taste. Panelists reported that the betalain content of MSFE notably improved the appearance and taste of the yogurt. Therefore, it is evident that incorporating MSFE in the yogurt enhanced the sensory attributes, surpassing the control sample.

## 4 Conclusion

MSFEs are valuable natural colorants for yogurt, enhancing both its sensory appeal and nutritional quality. This study demonstrated that incorporating MSFEs at various concentrations improved yogurt physicochemical properties, such as increasing total soluble solids, viscosity, and titratable acidity while decreasing pH and syneresis. The stability of color, along with its wide availability, abundance of betalain, and cost efficiency, allowed the MSFE to be incorporated into yogurt. This study aimed to incorporate MSFEs as a source of natural colorants in yogurt, revealing their greater acceptance of color, abundance of bioactive compounds, and potent antioxidant activity. Additionally, yogurt containing MSFEs presented greater TPC, TFC, and antioxidant activity than did the control yogurt. The highest concentration of MSFE resulted in the most significant improvement in color properties due to the high betalain content, despite slight degradation over time. The stability of the color across a broad pH range, combined with its cost‐effectiveness, natural origin, and health‐promoting properties, supports the feasibility of MSFEs as a viable alternative to synthetic colorants in the dairy industry. Further studies should investigate the applicability of MSFE in various fermented dairy products to assess its broader utility as a natural colorant and functional ingredient. Moreover, comprehensive safety assessments are essential to ensure the safe consumption of MSFE and identify any potential adverse effects. Furthermore, research should focus on strategies to minimize the slight inconsistency in color stability observed during the storage of the formulated yogurt. The use of sophisticated analytical techniques such as high‐performance liquid chromatography (HPLC) is highly recommended for accurate and precise quantification of betalain content and other bioactive compounds. Therefore, MSFE offers a sustainable, cost‐effective, and nutritionally beneficial alternative to artificial colorants in dairy products, with strong potential for consumer acceptance.

## Author Contributions

**Md. Akhtaruzzaman:** conceptualization (equal), funding acquisition (lead), project administration (lead), supervision (equal), writing – original draft (equal), writing – review and editing (equal). **Tanjim Ahmed:** conceptualization (equal), formal analysis (lead), investigation (equal), methodology (lead), writing – original draft (lead), writing – review and editing (equal). **Md. Rakibul Islam:** conceptualization (equal), data curation (equal), methodology (lead), supervision (equal), validation (equal), visualization (equal), writing – original draft (lead), writing – review and editing (lead). **Sharmin Akther:** conceptualization (equal), data curation (equal), software (equal), writing – review and editing (equal). **Md. Sajib Al Reza:** conceptualization (equal), validation (equal), visualization (equal), writing – review and editing (equal).

## Ethics Statement

Ethical approval for the involvement of human subjects in the sensory study for this research was granted by the Ethical Review Committee of the Faculty of Biological Science, Jashore University of Science and Technology, Jashore, Bangladesh, under reference number: ERC/FBST/JUST/2024–202.

## Conflicts of Interest

The authors declare no conflicts of interest.

## Data Availability

The data that support the findings of this study are available from the corresponding authors upon reasonable request.

## References

[fsn370710-bib-0001] Abdo, E. M. , H. M. M. Mansour , A. M. G. Darwish , et al. 2023. “Beetroot Stalk Extract as a Functional Colorant for Stirred Yogurt Beverages: Effect on Nutritional Value and Stability During Storage.” Fermentation 9, no. 10: 878. 10.3390/fermentation9100878.

[fsn370710-bib-0002] Adjei, M. L. , A. Boakye , G. Deku , et al. 2024. “Development of Yoghurt Incorporated With Beetroot Puree and Its Effect on the Physicochemical Properties and Consumer Acceptance.” Heliyon 10, no. 3: e25492. 10.1016/j.heliyon.2024.e25492. 38352778 PMC10862673

[fsn370710-bib-0003] Ahmed, M. , A. Ali , A. Sarfraz , Q. Hong , and H. Boran . 2022. “Effect of Freeze‐Drying on Apple Pomace and Pomegranate Peel Powders Used as a Source of Bioactive Ingredients for the Development of Functional Yogurt.” Journal of Food Quality 2022: 1–9. 10.1155/2022/3327401.

[fsn370710-bib-0004] Ahmed, T. , M. Akhtaruzzaman , M. R. Islam , M. Biswas , F. Kazi , and A. K. Das . 2025. “Beetroot Juice as a Natural Colorant in Functional Yogurt: Evaluation of Physicochemical, Bioactive, Microbial and Sensory Properties During Storage.” Journal of Food Measurement and Characterization 19: 4017–4033. 10.1007/s11694-025-03230-9.

[fsn370710-bib-0005] Alam, A. , M. Biswas , T. Ahmed , et al. 2023. “Effect of Ultrasound and Thermal Pasteurization on Physicochemical Properties and Antioxidant Activity of Juice Extracted From Ripe and Overripe Pineapple.” Food and Nutrition Sciences 14, no. 4: 300–314. 10.4236/fns.2023.144020.

[fsn370710-bib-0006] Alam, S. S. , S. Akther , M. R. Islam , M. R. Islam , and M. Akhtaruzzaman . 2023. “Effects of Ultrasound, Microwave, and Their Combined Treatments on the Shelf Life and Quality Characteristics of Fresh Litchi Juice.” Future Foods 8: 100254. 10.1016/j.fufo.2023.100254.

[fsn370710-bib-0007] Almusallam, I. A. , I. A. Mohamed Ahmed , A. Saleh , et al. 2021. “Potential of Date Palm Spikelet Extract as an Anti‐Oxidative Agent in Set‐Type Yogurt During Cold Storage.” CyTA Journal of Food 19, no. 1: 190–197. 10.1080/19476337.2021.1877826.

[fsn370710-bib-0008] Al‐Sahlany, S. T. G. , W. H. Khassaf , A. K. Niamah , and A. J. Abd Al‐Manhel . 2023. “Date Juice Addition to Bio‐Yogurt: The Effects on Physicochemical and Microbiological Properties During Storage, as Well as Blood Parameters in Vivo.” Journal of the Saudi Society of Agricultural Sciences 22, no. 2: 71–77. 10.1016/j.jssas.2022.06.005.

[fsn370710-bib-0009] Anuyahong, T. , C. Chusak , and S. Adisakwattana . 2020. “Incorporation of Anthocyanin‐Rich Riceberry Rice in Yogurts: Effect on Physicochemical Properties, Antioxidant Activity and in Vitro Gastrointestinal Digestion.” Lwt 129: 109571. 10.1016/j.lwt.2020.109571.

[fsn370710-bib-0010] Atwaa, E. H. , H. A. Badr , M. F. Ramadan , and D. I. Kabil . 2024. “Production of a Functional Yogurt Drink Enriched With Black Rice Beverage.” Journal of Food and Dairy Sciences 15, no. 4: 69–77. 10.21608/jfds.2024.282857.1155.

[fsn370710-bib-0011] Basavaraja, T. , A. Joshi , S. Sethi , et al. 2022. “Extraction Procedure of Betalains Pigments From Hardy Beetroot Matrix and Its Stabilization.” Journal of Food Processing and Preservation 46, no. 10: e16844. 10.1111/jfpp.16844.

[fsn370710-bib-0012] Basiony, M. , A. Saleh , R. Hassabo , and A. AL‐Fargah . 2023. “The Effect of Using Pomegranate and Strawberry Juices With Red Beet Puree on the Physicochemical, Microbial and Sensory Properties of Yoghurt.” Journal of Food Measurement and Characterization 17, no. 5: 5024–5033. 10.1007/s11694-023-01984-8.

[fsn370710-bib-0013] Bchir, B. , M. A. Bouaziz , C. Blecker , and H. Attia . 2020. “Physico‐Chemical, Antioxidant Activities, Textural, and Sensory Properties of Yoghurt Fortified With Different States and Rates of Pomegranate Seeds ( *Punica granatum* L.).” Journal of Texture Studies 51, no. 3: 475–487. 10.1111/jtxs.12500. 31785164

[fsn370710-bib-0014] Boonupara, T. , P. Udomkun , and P. Kajitvichyanukul . 2024. “Quantitative Analysis of Atrazine Impact on UAV‐Derived Multispectral Indices and Correlated Plant Pigment Alterations: A Heatmap Approach.” Agronomy 14, no. 4: 814. 10.3390/agronomy14040814.

[fsn370710-bib-0015] Buniowska‐Olejnik, M. , A. Mykhalevych , J. Urbański , I. Hadijeva , A. Berthold‐Pluta , and M. Banach . 2025. “Storage Quality and Antioxidant Properties of Yogurt Fortified With Highly Bioavailable Formula of Curcumin.” Lwt 223: 117798. 10.1016/j.lwt.2025.117798.

[fsn370710-bib-0016] Calva‐Estrada, S. J. , M. Jiménez‐Fernández , and E. Lugo‐Cervantes . 2022. “Betalains and Their Applications in Food: The Current State of Processing, Stability and Future Opportunities in the Industry.” Food Chemistry: Molecular Sciences 4: 100089. 10.1016/j.fochms.2022.100089. 35415668 PMC8991513

[fsn370710-bib-0017] Chan, S. M. , V. Y. Fong , S. Y. Koo , et al. 2022. “Antibacterial Activity of Selected Medicinal Plants From Malaysia.” Asia‐Pacific Journal of Science and Technology 27, no. 1: 1–10. 10.14456/apst.2022.2.

[fsn370710-bib-0018] Cho, W.‐Y. , S.‐H. Hwa , F. Yang , and C.‐H. Lee . 2020. “Quality Characteristics and Antioxidant Activity of Yogurt Containing Raw Omija and Sugared Omija During Storage.” Journal of Chemistry 2020: 1–7. 10.1155/2020/1274591.

[fsn370710-bib-0019] Cho, W.‐Y. , D.‐H. Kim , H.‐J. Lee , S.‐J. Yeon , and C.‐H. Lee . 2020. “Quality Characteristic and Antioxidant Activity of Yogurt Containing Olive Leaf Hot Water Extract.” CyTA Journal of Food 18, no. 1: 43–50. 10.1080/19476337.2019.1640797.

[fsn370710-bib-0020] CODEX Alimentarius Commission . 2003. Codex Standard for Fermented Milks. Codex Stan 243–2003. 2nd ed, 6–16. FAO/WHO. http://www.fao.org/3/i2085s/i2085s.pdf.

[fsn370710-bib-0021] Dhiman, A. , R. Suhag , D. S. Chauhan , D. Thakur , S. Chhikara , and P. K. Prabhakar . 2021. “Status of Beetroot Processing and Processed Products: Thermal and Emerging Technologies Intervention.” Trends in Food Science & Technology 114: 443–458. 10.1016/j.tifs.2021.05.042.

[fsn370710-bib-0022] Dias, P. G. I. , J. W. A. Sajiwanie , and R. M. U. S. K. Rathnayaka . 2020. “Formulation and Development of Composite Fruit Peel Powder Incorporated Fat and Sugar‐Free Probiotic Set Yogurt.” GSC Biological and Pharmaceutical Sciences 11, no. 1: 93–99. 10.30574/gscbps.2020.11.1.0084.

[fsn370710-bib-0023] El‐Messery, T. M. , E. Aly , R. López‐Nicolas , T. Sánchez‐Moya , and G. Ros . 2021. “Bioaccessibility and Antioxidant Activity of PCL‐Microencapsulated Olive Leaves Polyphenols and Its Application in Yogurt.” Journal of Food Science 86, no. 10: 4303–4315. 10.1111/1750-3841.15893. 34496055

[fsn370710-bib-0024] Fernando, G. S. N. , K. Wood , E. H. Papaioannou , L. J. Marshall , N. N. Sergeeva , and C. Boesch . 2021. “Application of an Ultrasound‐Assisted Extraction Method to Recover Betalains and Polyphenols From Red Beetroot Waste.” ACS Sustainable Chemistry & Engineering 9, no. 26: 8736–8747. 10.1021/acssuschemeng.1c01203.

[fsn370710-bib-0025] Flores‐Mancha, M. A. , M. G. Ruíz‐Gutiérrez , A. L. Rentería‐Monterrubio , et al. 2021. “Stirred Yogurt Added With Beetroot Extracts as an Antioxidant Source: Rheological, Sensory, and Physicochemical Characteristics.” Journal of Food Processing and Preservation 45, no. 7: e15628. 10.1111/jfpp.15628.

[fsn370710-bib-0026] Flores‐Mancha, M. A. , M. G. Ruíz‐Gutiérrez , R. Sánchez‐Vega , E. Santellano‐Estrada , and A. Chávez‐Martínez . 2021. “Effect of Encapsulated Beet Extracts ( *Beta vulgaris* ) Added to Yogurt on the Physicochemical Characteristics and Antioxidant Activity.” Molecules 26, no. 16: 4768. 10.3390/molecules26164768. 34443359 PMC8401705

[fsn370710-bib-0027] Fu, Y. , J. Shi , S.‐Y. Xie , T.‐Y. Zhang , O. P. Soladoye , and R. E. Aluko . 2020. “Red Beetroot Betalains: Perspectives on Extraction, Processing, and Potential Health Benefits.” Journal of Agricultural and Food Chemistry 68, no. 42: 11595–11611. 10.1021/acs.jafc.0c04241. 33040529

[fsn370710-bib-0028] Gengatharan, A. , G. Dykes , and W. S. Choo . 2021. “Betacyanins From *Hylocereus polyrhizus* : Pectinase‐Assisted Extraction and Application as a Natural Food Colourant in Ice Cream.” Journal of Food Science and Technology 58: 1401–1410. 10.1007/s13197-020-04651-8. 33746268 PMC7925792

[fsn370710-bib-0029] Guneser, O. 2021. “Kinetic Modelling of Betalain Stability and Color Changes in Yogurt During Storage.” Polish Journal of Food and Nutrition Sciences 71: 135–145. 10.31883/pjfns/134393.

[fsn370710-bib-0030] Halladj, F. , H. Amellal‐Chibane , R. Aitfella‐Lahlou , M. A. Bourai , and A. Tigrine . 2022. “Effect of Red Beet Cooking Water on Yoghurt's Physico‐Chemical, Textural and Antioxidant Characteristics.” Food Science and Technology International 30, no. 1: 85–93. 10.1177/10820132221137386. 36377357

[fsn370710-bib-0031] Hasan, M. M. , M. R. Islam , A. R. Haque , M. R. Kabir , K. J. Khushe , and S. K. Hasan . 2024. “Trends and Challenges of Fruit By‐Products Utilization: Insights Into Safety, Sensory, and Benefits of the Use for the Development of Innovative Healthy Food: A Review.” Bioresources and Bioprocessing 11, no. 1: 10. 10.1186/s40643-023-00722-8. 38647952 PMC10991904

[fsn370710-bib-0032] Hasan, S. M. K. , M. R. Islam , M. R. kabir , M. M. Rahman , M. R. Islum , and M. M. Esha . 2024. “Exploring the Nutraceutical Potential: Evaluating the Nutritional and Bioactive Functions of Five Pomelo Fruit Varieties in Bangladesh.” Heliyon 10, no. 11: e31786. 10.1016/j.heliyon.2024.e31786. 38845880 PMC11153172

[fsn370710-bib-0033] Hasan, S. M. K. , M. R. Kabir , M. R. Kabir , M. R. Islam , M. J. Akhter , and J. Y. Moury . 2022. “Proximate Composition, Minerals, Phytochemicals, and Functional Activities of Jujube Fruits Grown in Bangladesh.” Journal of Agriculture and Food Research 8: 100302. 10.1016/j.jafr.2022.100302.

[fsn370710-bib-0034] Ikram, A. , S. Qasim Raza , F. Saeed , et al. 2021. “Effect of Adding *Aloe vera* Jell on the Quality and Sensory Properties of Yogurt.” Food Science & Nutrition 9, no. 1: 480–488. 10.1002/fsn3.2017. 33473309 PMC7802546

[fsn370710-bib-0035] Islam, M. R. , M. M. H. Biswas , M. K. H. Esham , P. Roy , M. R. khan , and S. M. K. Hasan . 2023. “Jackfruit ( *Artocarpus heterophyllus* ) by‐Products a Novel Source of Pectin: Studies on Physicochemical Characterization and Its Application in Soup Formulation as a Thickener.” Food Chemistry Advances 2: 100273. 10.1016/j.focha.2023.100273.

[fsn370710-bib-0036] Islam, T. , M. R. Repon , T. Islam , Z. Sarwar , and M. M. Rahman . 2023. “Impact of Textile Dyes on Health and Ecosystem: A Review of Structure, Causes, and Potential Solutions.” Environmental Science and Pollution Research 30, no. 4: 9207–9242. 10.1007/s11356-022-24398-3. 36459315

[fsn370710-bib-0037] Jouki, M. , N. Khazaei , S. Rashidi‐Alavijeh , and S. Ahmadi . 2021. “Encapsulation of *Lactobacillus casei* in Quince Seed Gum‐Alginate Beads to Produce a Functional Synbiotic Drink Powder by Agro‐Industrial By‐Products and Freeze‐Drying.” Food Hydrocolloids 120: 106895. 10.1016/j.foodhyd.2021.106895.

[fsn370710-bib-0039] Kabir, M. R. , M. M. Hasan , M. R. Islam , A. R. Haque , and S. M. K. Hasan . 2021. “Formulation of Yogurt With Banana Peel Extracts to Enhance Storability and Bioactive Properties.” Journal of Food Processing and Preservation 45, no. 3: e15191. 10.1111/jfpp.15191.

[fsn370710-bib-0038] Kabir, M. R. , S. M. K. Hasan , M. R. Islam , and M. Ahmed . 2024. “Development of Functional Noodles by Encapsulating Mango Peel Powder as a Source of Bioactive Compounds.” Heliyon 10, no. 1: e24061. 10.1016/j.heliyon.2024.e24061. 38230233 PMC10789624

[fsn370710-bib-0040] Kim, S. Y. , O. Hyeonbin , P. Lee , and Y.‐S. Kim . 2020. “The Quality Characteristics, Antioxidant Activity, and Sensory Evaluation of Reduced‐Fat Yogurt and Nonfat Yogurt Supplemented With Basil Seed Gum as a Fat Substitute.” Journal of Dairy Science 103, no. 2: 1324–1336. 10.3168/jds.2019-17117. 31785875

[fsn370710-bib-0041] Kozioł, Ł. , M. Knap , K. Sutor‐Świeży , et al. 2024. “Identification and Reactivity of Pigments in Prominent Vegetable Leaves of *Basella alba* L. Var. “Rubra” (Malabar Spinach).” Food Chemistry 445: 138714. 10.1016/j.foodchem.2024.138714. 38394904

[fsn370710-bib-0042] Lazăr, S. , O. E. Constantin , G. Horincar , et al. 2022. “Beetroot By‐Product as a Functional Ingredient for Obtaining Value‐Added Mayonnaise.” Processes 10, no. 2: 227. 10.3390/pr10020227.

[fsn370710-bib-0043] Liliana, C. , and N. Oana‐Viorela . 2020. “Red Beetroot: Composition and Health Effects ‐ A Review.” Journal of Nutritional Medicine and Diet Care 6, no. 1: 1–9. 10.23937/2572-3278.1510043.

[fsn370710-bib-0044] Linares, G. , and M. L. Rojas . 2022. “Ultrasound‐Assisted Extraction of Natural Pigments From Food Processing By‐Products: A Review.” Frontiers in Nutrition 9: 891462. 10.3389/fnut.2022.891462. 35685880 PMC9171369

[fsn370710-bib-0045] Lugo‐Zarate, L. , N. d. S. Cruz‐Cansino , E. Ramírez‐Moreno , et al. 2021. “Evaluation of Physicochemical, Microbiological, and Antioxidant Properties of a Drinkable Yogurt Added With Ultrasonicated Purple Cactus Pear ( *Opuntia ficus‐indica* ) Juice Powder.” Journal of Food Processing and Preservation 45, no. 9: e15720. 10.1111/jfpp.15720.

[fsn370710-bib-0046] Miguel, M. G. 2018. “Betalains in Some Species of the Amaranthaceae Family: A Review.” Antioxidants 7, no. 4: 53. 10.3390/antiox7040053. 29617324 PMC5946119

[fsn370710-bib-0047] Muflihah, Y. M. , G. Gollavelli , and Y.‐C. Ling . 2021. “Correlation Study of Antioxidant Activity With Phenolic and Flavonoid Compounds in 12 Indonesian Indigenous Herbs.” Antioxidants 10, no. 10: 1530. 10.3390/antiox10101530. 34679665 PMC8533117

[fsn370710-bib-0048] Mukta, N. A. , A. I. Mustafa , A. K. M. S. Inam , and M. Hasan . 2021. “Physicochemical, Sensory and Microbial Assessment of Newly Formulated and Fortified Yogurt.” Carpathian Journal of Food Science and Technology 13, no. 2: 64–73. 10.34302/crpjfst/2021.13.2.6.

[fsn370710-bib-0049] Nabi, B. G. , K. Mukhtar , W. Ahmed , et al. 2023. “Natural Pigments: Anthocyanins, Carotenoids, Chlorophylls, and Betalains as Colorants in Food Products.” Food Bioscience 52: 102403. 10.1016/j.fbio.2023.102403.

[fsn370710-bib-0050] Navarro, F. , E. Apablaza , J. C. Carmona , and C. Sáenz . 2022. “Characterization, Stability and Application in Yogurt of a Coloring Food From *Opuntia robusta* Fruits.” Acta Horticulturae 1343: 395–400. 10.17660/ActaHortic.2022.1343.50.

[fsn370710-bib-0051] Nirmal, N. P. , R. Mereddy , and S. Maqsood . 2021. “Recent Developments in Emerging Technologies for Beetroot Pigment Extraction and Its Food Applications.” Food Chemistry 356: 129611. 10.1016/j.foodchem.2021.129611. 33838608

[fsn370710-bib-0052] Nur, M. A. , M. Islam , S. Biswas , et al. 2023. “Determination of Biological Activities of Malabar Spinach ( *Basella alba* ) Fruit Extracts and Molecular Docking Against COX‐II Enzyme.” Heliyon 9, no. 11: e21568. 10.1016/j.heliyon.2023.e21568. 38027774 PMC10663853

[fsn370710-bib-0053] Nur, M. A. , M. Khan , M. A. Satter , M. M. Rahman , M. Jashim uddin , and M. Z. Amin . 2023. “Assessment of Physicochemical Properties, Nutrient Contents and Colorant Stability of the Two Varieties of Malabar Spinach ( *Basella alba* L.) Fruits.” Biocatalysis and Agricultural Biotechnology 51: 102746. 10.1016/j.bcab.2023.102746.

[fsn370710-bib-0054] Olas, B. , J. Białecki , K. Urbańska , and M. Bryś . 2021. “The Effects of Natural and Synthetic Blue Dyes on Human Health: A Review of Current Knowledge and Therapeutic Perspectives.” Advances in Nutrition 12, no. 6: 2301–2311. 10.1093/advances/nmab081. 34245145 PMC8634323

[fsn370710-bib-0055] Phimolsiripol, Y. , S. Buadoktoom , P. Leelapornpisid , et al. 2021. “Shelf Life Extension of Chilled Pork by Optimal Ultrasonicated Ceylon Spinach ( *Basella alba* ) Extracts: Physicochemical and Microbial Properties.” Food 10, no. 6: 1241. 10.3390/foods10061241. PMC8227812 34072425

[fsn370710-bib-0056] Rahmani, F. , H. Gandomi , N. Noori , A. Faraki , and M. Farzaneh . 2021. “Microbial, Physiochemical and Functional Properties of Probiotic Yogurt Containing *Lactobacillus Acidophilus* and *Bifidobacterium bifidum* Enriched by Green Tea Aqueous Extract.” Food Science & Nutrition 9, no. 10: 5536–5545. 10.1002/fsn3.2512. 34646523 PMC8498050

[fsn370710-bib-0057] Rocha, F. , C. S. Marques , L. S. de Sousa , et al. 2022. “Betalains Nanodispersions: Effects on Betalains Stability and on Rheological Properties of Greek Yogurt.” Food Research International 159: 111583. 10.1016/j.foodres.2022.111583. 35940758

[fsn370710-bib-0058] Saberi, M. , S. Saremnezhad , M. Soltani , and A. Faraji . 2023. “Functional Stirred Yogurt Manufactured Using Co‐Microencapsulated or Free Forms of Grape Pomace and Flaxseed Oil as Bioactive Ingredients: Physicochemical, Antioxidant, Rheological, Microstructural, and Sensory Properties.” Food Science & Nutrition 11, no. 7: 3989–4001. 10.1002/fsn3.3385. 37457195 PMC10345739

[fsn370710-bib-0059] Sadowska‐Bartosz, I. , and G. Bartosz . 2021. “Biological Properties and Applications of Betalains.” Molecules 26, no. 9: 2520. 10.3390/molecules26092520. 33925891 PMC8123435

[fsn370710-bib-0060] Sagar, V. , Pragya , R. Bhardwaj , et al. 2022. “The Inheritance of Betalain Pigmentation in *Basella alba* L.” South African Journal of Botany 145: 360–369. 10.1016/j.sajb.2022.01.033.

[fsn370710-bib-0061] Schneider‐Teixeira, A. , A. D. Molina‐García , I. Alvarez , M. D. Staffolo , and L. Deladino . 2022. “Application of Betacyanins Pigments From *Alternanthera brasiliana* as Yogurt Colorant.” Lwt 159: 113237. 10.1016/j.lwt.2022.113237.

[fsn370710-bib-0062] Shraim, A. M. , T. A. Ahmed , M. M. Rahman , and Y. M. Hijji . 2021. “Determination of Total Flavonoid Content by Aluminum Chloride Assay: A Critical Evaluation.” Lwt 150: 111932. 10.1016/j.lwt.2021.111932.

[fsn370710-bib-0063] Sigwela, V. N. , M. de Wit , A. Du Toit , and A. Hugo . 2022. “Application of Betalain Extracts as Colouring Foods to Food Products.” Acta Horticulturae 1343: 463–472. 10.17660/ActaHortic.2022.1343.58.

[fsn370710-bib-0064] Skalicky, M. , J. Kubes , H. Shokoofeh , M. Tahjib‐Ul‐Arif , P. Vachova , and V. Hejnak . 2020. “Betacyanins and Betaxanthins in Cultivated Varieties of *Beta vulgaris* L. Compared to Weed Beets.” Molecules 25, no. 22: 5395. 10.3390/molecules25225395. 33218115 PMC7698878

[fsn370710-bib-0065] Stuivenberg, G. A. , J. A. Chmiel , P. P. Akouris , et al. 2023. “Supplementing Yogurt With Probiotic Bifidobacteria to Counter Chronic Kidney Disease.” Fermentation 9, no. 4: 391. 10.3390/fermentation9040391.

[fsn370710-bib-0066] Sutakwa, A. , L. S. Nadia , and S. Suharman . 2021. “Addition of Blue Pea Flower ( *Clitoria ternatea* L.) Extract Increase Antioxidant Activity in Yogurt From Various Types of Milk.” Jurnal Agercolere 3, no. 1: 31–37. 10.37195/jac.v3i1.123.

[fsn370710-bib-0067] Sutor‐Świeży, K. , R. Górska , A. Kumorkiewicz‐Jamro , et al. 2024. “ *Basella alba* L. (Malabar Spinach) as an Abundant Source of Betacyanins: Identification, Stability, and Bioactivity Studies on Natural and Processed Fruit Pigments.” Journal of Agricultural and Food Chemistry 72, no. 6: 2943–2962. 10.1021/acs.jafc.3c06225. 38301126 PMC10870984

[fsn370710-bib-0068] Thivya, P. , N. Bhanu Prakash Reddy , K. Bhosale Yuvraj , and V. R. Sinija . 2023. “Recent Advances and Perspectives for Effective Utilization of Onion Solid Waste in Food Packaging: A Critical Review.” Reviews in Environmental Science and Bio/Technology 22, no. 1: 29–53. 10.1007/s11157-022-09642-z.

[fsn370710-bib-0069] Wijesekara, A. , V. Weerasingha , S. Jayarathna , and H. Priyashantha . 2022. “Quality Parameters of Natural Phenolics and Its Impact on Physicochemical, Microbiological, and Sensory Quality Attributes of Probiotic Stirred Yogurt During the Storage.” Food Chemistry: X 14: 100332. 10.1016/j.fochx.2022.100332. 35634218 PMC9130075

[fsn370710-bib-0070] Ye, Y. , P. Li , J. Zhou , J. He , and J. Cai . 2022. “The Improvement of Sensory and Bioactive Properties of Yogurt With the Introduction of Tartary Buckwheat.” Food 11, no. 12: 1774. 10.3390/foods11121774. PMC9222765 35741972

[fsn370710-bib-0071] Yildirim‐Elikoglu, S. , and Y. K. Erdem . 2018. “Interactions Between Milk Proteins and Polyphenols: Binding Mechanisms, Related Changes, and the Future Trends in the Dairy Industry.” Food Reviews International 34, no. 7: 665–697. 10.1080/87559129.2017.1377225.

